# Acoustic–phonetic and auditory mechanisms of adaptation in the perception of sibilant fricatives

**DOI:** 10.3758/s13414-019-01894-2

**Published:** 2019-12-24

**Authors:** Eleanor Chodroff, Colin Wilson

**Affiliations:** 1 Department of Language and Linguistic Science, University of York, Heslington, York, YO10 5DD UK; 2 Department of Cognitive Science, Johns Hopkins University, 3400 N. Charles St., Baltimore, MD 21218 USA

**Keywords:** Speech perception, Perceptual learning, Audition

## Abstract

Listeners are highly proficient at adapting to contextual variation when perceiving speech. In the present study, we examined the effects of brief speech and nonspeech contexts on the perception of sibilant fricatives. We explored three theoretically motivated accounts of contextual adaptation, based on phonetic cue calibration, phonetic covariation, and auditory contrast. Under the *cue calibration* account, listeners adapt by estimating a talker-specific average for each phonetic cue or dimension; under the *cue covariation* account, listeners adapt by exploiting consistencies in how the realization of speech sounds varies across talkers; under the *auditory contrast* account, adaptation results from (partial) masking of spectral components that are shared by adjacent stimuli. The spectral center of gravity, a phonetic cue to fricative identity, was manipulated for several types of context sound: /z/-initial syllables, /v/-initial syllables, and white noise matched in long-term average spectrum (LTAS) to the /z/-initial stimuli. Listeners’ perception of the /s/–/ʃ/ contrast was significantly influenced by /z/-initial syllables and LTAS-matched white noise stimuli, but not by /v/-initial syllables. No significant difference in adaptation was observed between exposure to /z/-initial syllables and matched white noise stimuli, and speech did not have a considerable advantage over noise when the two were presented consecutively within a context. The pattern of findings is most consistent with the auditory contrast account of short-term perceptual adaptation. The cue covariation account makes accurate predictions for speech contexts, but not for nonspeech contexts or for the absence of a speech-versus-nonspeech difference.

## Introduction

The realization of a speech sound can vary substantially according to contextual factors such as neighboring speech sounds, global factors like speaking rate, and talker characteristics, including physiology, dialect, and idiosyncratic factors (e.g., Byrd, 1992; Delattre, Liberman, & Cooper, 1955; Johnson & Beckman, 1997; Liberman, Cooper, Shankweiler, & Studdert-Kennedy, 1967; Miller, Green, & Reeves, 1986; Nolan, 1983; Peterson & Barney, 1952). Listeners can compensate for such factors by rapidly adapting their perception of speech to the context in which it is heard. For example, a stop consonant that is ambiguous between /d/ and /g/ is more likely to be perceived as /g/ following the syllable /al/, and as /d/ following the syllable /aɹ/ (Mann, 1980). Listeners are also more likely to identify a stop consonant as voiceless when it is preceded by a sentence with a faster speaking rate, relative to one spoken at a slower rate (Miller et al., 1986). In addition, listeners have been shown to adapt to talker-specific realizations of various speech sounds, including vowels (e.g., Maye, Aslin, & Tanenhaus, 2008), stop consonants (e.g., Allen & Miller, 2004; Theodore & Miller, 2010), and fricatives (e.g., Norris, McQueen, & Cutler, 2003).

In the present study, we examined the effects of brief speech and nonspeech contexts on the perception of sibilant fricatives. Fricatives such as /s/ vary in their spectral properties across phonetic contexts (e.g., Jongman, Wayland, & Wong, 2000; Soli, 1981) and different talkers or talker groups (e.g., Flipsen, Shriberg, Weismer, Karlsson, & McSweeny, 1999; Jongman et al., 2000; Newman, Clouse, & Burnham, 2001). Listeners adapt to such context- and talker-specific realizations. For example, listeners shift the perceptual boundary between /s/ and /f/ according to whether a talker produced words with a relatively /s/-like /f/ or a /f/-like /s/, and this adaptation is specific to the particular talker (Eisner & McQueen, 2005; Norris et al., 2003; see also Kraljic & Samuel, 2007). Such adaptation effects can persist over several hours outside of the laboratory (Eisner & McQueen, 2006; Kraljic & Samuel, 2005).

Previous studies have also shown that listeners generalize adaptation of fricative perception to words (McQueen, Cutler, & Norris, 2006) and to fricatives that have been withheld from exposure (Durvasula & Nelson, 2018). For example, listeners exposed to an /s/-like /f/ in real lexical items were more likely to indicate that the talker also had more /z/-like /v/s. Existing findings about fricative adaptation are therefore not reducible to mechanisms that operate entirely on individual lexical items or sounds (e.g., the storage of word-specific phonetic exemplars) or to brief changes in auditory sensitivity (e.g., as in forward spectral masking [Houtgast, 1974; Moore & Glasberg, 1981] or auditory sensory memory [Cowan, 1984; Nees, 2016; Neisser, 1967]).

Many conceivable representations and processes could underlie adaptive speech perception (see Samuel & Kraljic, 2009, for a review). In this article, we compare three mechanisms that make contrasting predictions about short-term adaptation effects on the perception of sibilant fricatives (i.e., the /s/–/ʃ/ contrast), presenting results that shed light on adaptation mechanisms more generally. In the following sections, we review the mechanisms that are explored in the present article: *phonetic cue calibration*, *phonetic cue covariation*, and *auditory contrast*. The first two mechanisms involve adaptation to sublexical representations, whereas the third mechanism is based on general auditory processes. To distinguish among the adaptation mechanisms, we manipulated the spectral center of gravity (COG), a known phonetic cue to fricative perception, for four types of contexts that preceded /s/–/ʃ/ categorization: /z/-initial syllables, /v/-initial syllables, white noise that was matched in long-term average spectrum (LTAS) to the /z/-initial stimuli, and alternating, opposing presentations of the /z/-initial syllables and white noise. The final experiment was designed to directly test the relative strength of LTAS-matched linguistic and nonlinguistic stimuli.

### Cue calibration

Every speech sound has a number of acoustic correlates or *cues* that signal its presence and distinguish it from other sounds (Wright, 2004). Determining which acoustic cues are perceptually valid is a long-standing pursuit in speech research, and fricative identification is no exception to this inquiry. Listeners are highly sensitive to differences in spectral shape in the perceptual identification and discrimination of fricative categories (e.g., Harris, 1958; Jongman et al., 2000; McMurray & Jongman, 2011). Spectral shape can be partially summarized by *center of gravity* (COG): the magnitude-weighted average of the frequencies that are present in the fricative spectrum (e.g., Forrest, Weismer, Milenkovic, & Dougall, 1988).

COG can be used to distinguish sibilant fricatives with different places of articulation (/s z/ vs. /ʃ ʒ/); nonsibilants (/f v θ ð/) tend to have a broad and flat distribution of energy with overall higher spectral peaks relative to sibilants, but with COGs medial to the alveolar (/s z/) and postalveolar (/ʃ ʒ/) sibilants (e.g., Ali, Van der Spiegel, & Mueller, 2001; Forrest et al., 1988; Hughes & Halle, 1956; Jongman et al., 2000). Critically, the characteristic COG of a fricative can vary according to the phonetic context (e.g., the value for /s/ is lower before rounded vowels such as /u/ than before unrounded vowel such as /i/; Jongman et al., 2000; Soli, 1981; Yu, 2019), speech style (e.g., /s/ has a lower value in casual than in careful speech; Maniwa, Jongman, & Wade, 2009), talker gender (e.g., /s/ is lower on average for male than for female speakers of American English; Flipsen et al., 1999; Fuchs & Toda, 2010), and even across individual talkers of the same gender (e.g., Newman et al., 2001).

According to the cue calibration mechanism, adaptive speech perception makes use of talker-specific (and context-specific) statistics for COG and other cues. Support for this mechanism of adaptation is largely derived from quantitative models of extrinsic normalization that employ either mean subtraction or *z-*scoring across multiple phonetic categories for a single phonetic cue (e.g., for vowels, Lobanov, 1971; Nearey, 1978; for fricatives, McMurray & Jongman, 2011). For example, the probabilistic “sliding template” model of vowel normalization in Nearey and Assmann (2007) estimates talker-specific vowel spaces using a single offset from a template of vowel categories specified in the log F1×F2 space. In modeling perceptual categorization of American English fricatives, McMurray and Jongman (2011) found that categorization accuracy increased by 10% when their model included talker- and context-specific offsets for each fricative cue. Critically, the talker-specific offset for each cue was calculated using production data for that talker from *all* fricative categories together (/f v θ ð s z ʃ ʒ/).

This mechanism predicts generalization of talker-specific properties from one sound (e.g., /z/) to another (e.g., /s/ or /ʃ/), as follows. The listener could directly estimate the talker-specific COG average for /z/  from exposure items, compare this to the internally represented population mean for the same fricative (), and estimate the talker-specific effect on COG by subtraction . Expectations about how the same talker would realize /s/, /ʃ/, or indeed any other fricative on the COG dimension would then be derived by adding the same effect to their population means (e.g., ). For example, a talker with a COG for /z/ that is high relative to the population average would have a positive offset, and correspondingly higher expected means for /s/ and /ʃ/; this would in turn shift the /s/–/ʃ/ category boundary toward the /s/ end of a fixed continuum.

This account makes the prediction that, because perceptual adaptation involves estimation of a single talker offset for each cue, adaptation should equally affect all sounds bearing that cue. In particular, the inference that a talker has a relatively high-COG /s/ could be obtained from exposure to a high-COG /v/ just as well as to a high-COG /z/ as information from both /v/ and /z/ contribute to estimation of the talker-specific cue. Furthermore, since this mechanism is based on speech cues, it should not be engaged by nonspeech sounds. If adaptation effects on fricative perception are observed from nonspeech exposure, then a separate mechanism would have to be involved.

### Cue covariation

The phonetic cue covariation account of adaptation parallels cue calibration insofar as it involves adapting to talker-specific values of sounds on phonetic cue dimensions. It does not assume, however, that the talker-specific values of all sounds vary uniformly along a given dimension; instead, adaptation proceeds on the basis of empirical patterns of covariation or *mutual predictability* among speech sounds across talkers. Based on prior knowledge of population patterns, listeners could use evidence about one speech sound to infer properties of a second highly correlated sound, even without direct exposure to the second category. Critically, the strength of covariation for a given phonetic cue dimension will differ among speech sounds.

**Fig. 1:**
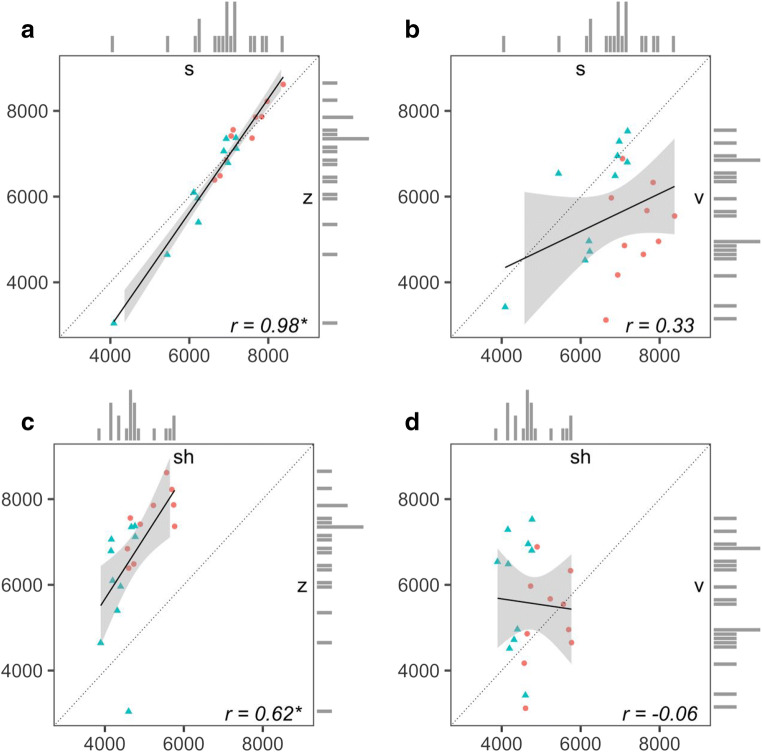
Variation and covariation of talker-specific center-of-gravity (COG) means (in hertz) between (a) /s/ and /z/, (b) /s/ and /v/, (c) /ʃ/ and /z/, and (d) ⁠/ʃ/ and /v/ in the Jongman et al. (2000) data. The COG means for male talkers are shown as triangles, and those for female talkers as circles in a different color. Pearson correlation coefficients are displayed in the lower right corner; an asterisk indicates that the correlation was significant at *p* < .01. Gray shading reflects the local confidence interval around the best-fit linear regression line; the dashed line reflects the line of equality; and the marginal histograms indicate the range of talker variation for each segment separately

In the case of fricatives, the talker-specific mean COG for /s/ is almost perfectly correlated with the corresponding mean COG for /z/ (*r* =.98, *p* < .01), whereas the correlation of talker mean COGs between /s/ and /v/ is relatively weak (*r* =.33, *p* = *.*16). The correlation between /ʃ/ and /z/ is also strong relative to that between /ʃ/ and /v/ (/ʃ/–/z/, *r* =.62, *p* < .01; /ʃ/–/v/, *r* = – .06, *p* = *.*80). These correlations were computed on data from 20 speakers of American English, reported in Jongman et al. (2000) and McMurray and Jongman (2011). Variation and covariation of these talker- and fricative-specific means are shown in Fig. 1.^1^

The clear prediction is that a talker’s characteristic COG should be generalized from /z/ to /s/ (and /ʃ/), but *not* from /v/ to the sibilants, given the empirical differences in cross-talker correlations. A listener could estimate the talker-specific COG mean for  directly from exposure. Together with the empirical correlations  and to some extent  (or, similarly, the linear fits of COG means across talkers), the estimate of  would allow the listener to project talker-specific means for the other two sibilants. Because the correlations between /s/ and /z/ and between /ʃ/ and /z/ are positive and strong, a talker who is observed to have a high COG for /z/ is also likely to have a high COG for /s/ and /ʃ/. Therefore, exposure to a talker with a high-COG /z/ should shift the /s/–/ʃ/ category boundary toward /s/, with the opposite shift being expected from exposure to a talker with a low-COG /z/.

Although this account makes the same prediction as the cue calibration account for generalization from /z/ to /s/, the cue covariation account departs from the cue calibration account in its prediction for /v/ and /s/. Though COG serves as a phonetic cue to both /v/ and /s/, the empirical relationship between a talker’s realization of /v/ and /s/ along this dimension is weak relative to the correlation between /z/ and /s/. Little adaptation to /s/ or /ʃ/ is therefore predicted from exposure to a talker’s realization of /v/. Similar to cue calibration, however, this account also involves adaptation mechanisms based solely on the properties of speech. Any influence of nonspeech stimuli on fricative perception would indicate an alternative mechanism of adaptation.

Importantly, the cue covariation account could easily be reformulated as a form of distinctive feature-based adaptation under a certain set of assumptions. Such an account has been investigated in previous studies of perceptual adaptation (e.g., Chládková, Boersma, & Benders, 2015; Chládková, Podlipský, & Chionidou, 2017; Durvasula & Nelson, 2018; Mitterer, Cho, & Kim, 2016; Reinisch, Wozny, Mitterer, & Holt, 2014). First, it must be assumed that the phonetic dimension along which listeners adapt strongly reflects an underlying feature value. In the present case, COG primarily reflects place of articulation and therefore a particular value of a place of articulation feature such as [anterior]. (Critical in this argument is not the name of the distinctive feature, but rather the fact that /s/ and /z/ share a feature specification.) Because /s/ and /z/ are both specified [+anterior], the phonetic realization of that feature may be *uniform* within a talker, which would give rise to strong covariation due to underlying identity (see Chodroff & Wilson, 2017, for how this may apply in stop consonants). Because /v/ and /s/ do not share a place of articulation, the COGs would reflect different distinctive feature values, and strong covariation may not exist (as empirically it does not).

Although the relationship between distinctive features and their acoustic expression is a matter of intense study and debate (Cole & Scott, 1974; Jakobson, Fant, & Halle, 1951; Marslen-Wilson & Warren, 1994; Stevens & Blumstein, 1978; Stevens & Keyser, 1989), any feature-based adaptation account necessarily assumes covariation. If a given feature could be expressed in completely independent ways across segments, there would be no basis for generalizing what is learned about the acoustic/auditory realization of one segment to another with the same specification.

### Auditory contrast

The third and final alternative we consider is an auditory mechanism of adaptation related to contrast enhancement. Adaptation of this type is a general sensory phenomenon, having demonstrated effects not only in audition (Dias, Cook, & Rosenblum, 2016; Roberts & Summerfield, 1981; Summerfield, Sidwell, & Nelson, 1987), but also in vision (e.g., Blakemore & Campbell, 1969; Hess, Dakin, & Field, 1998; Pantle & Sekuler, 1968) and olfaction (e.g., Cleland & Sethupathy, 2006). Perceptual sensitivity is enhanced to aspects of a stimulus following exposure to a context with contrastive properties, and perceptual sensitivity is diminished following exposure to a context with shared properties. For example, if a spectral peak exists within a particular frequency band in two adjacent stimuli, it will be perceived as weaker in the second stimulus (e.g., Diehl & Kluender, 1989; Houtgast, 1974; Kluender, Coady, & Kiefte, 2003; Yang, Luo, & Nehorai, 2003).

Although adaptation in the perception of fricatives has been shown to occur after repeated exposures and in a way that persists over relatively long timescales (e.g., Eisner & McQueen, 2005, 2006), adaptation generally can occur after brief exposure (e.g., Ladefoged & Broadbent, 1957) and, critically, can be induced by nonspeech auditory precursors (e.g., Holt, 2005, 2006; Kingston et al., 2014; Laing, Liu, Lotto, & Holt, 2012; Lotto & Kluender, 1998; Watkins & Makin, 1994, 1996). For instance, listeners reported more /ga/ than /da/ percepts for stimuli varying in F3 when the preceding syllable was /al/ than when it was /aɹ/ (Mann, 1980). A comparable effect was also observed when /al/ and /aɹ/ were replaced respectively with a sine wave tone in a high or low F3 region, which mimicked the distinctive concentration of energy in /l/ and /ɹ/ (Lotto & Kluender, 1998). Short-term perceptual adaptation and generalization may therefore be related to general auditory effects like spectral contrast, as opposed to a speech-specific adaptation mechanism.

For spectral contrast to arise, the manipulation of the precursor must be in a frequency range relevant for categorization of the following stimulus. Replicating Lotto and Kluender (1998), Laing et al. (2012) found that the effect of a sine-wave tone series with an F3 manipulation also influenced the perception of /d/ and /g/; however, when the tone series varied within the F1 frequency range, there was no significant effect on /d/–/g/ categorization.

**Fig. 2:**
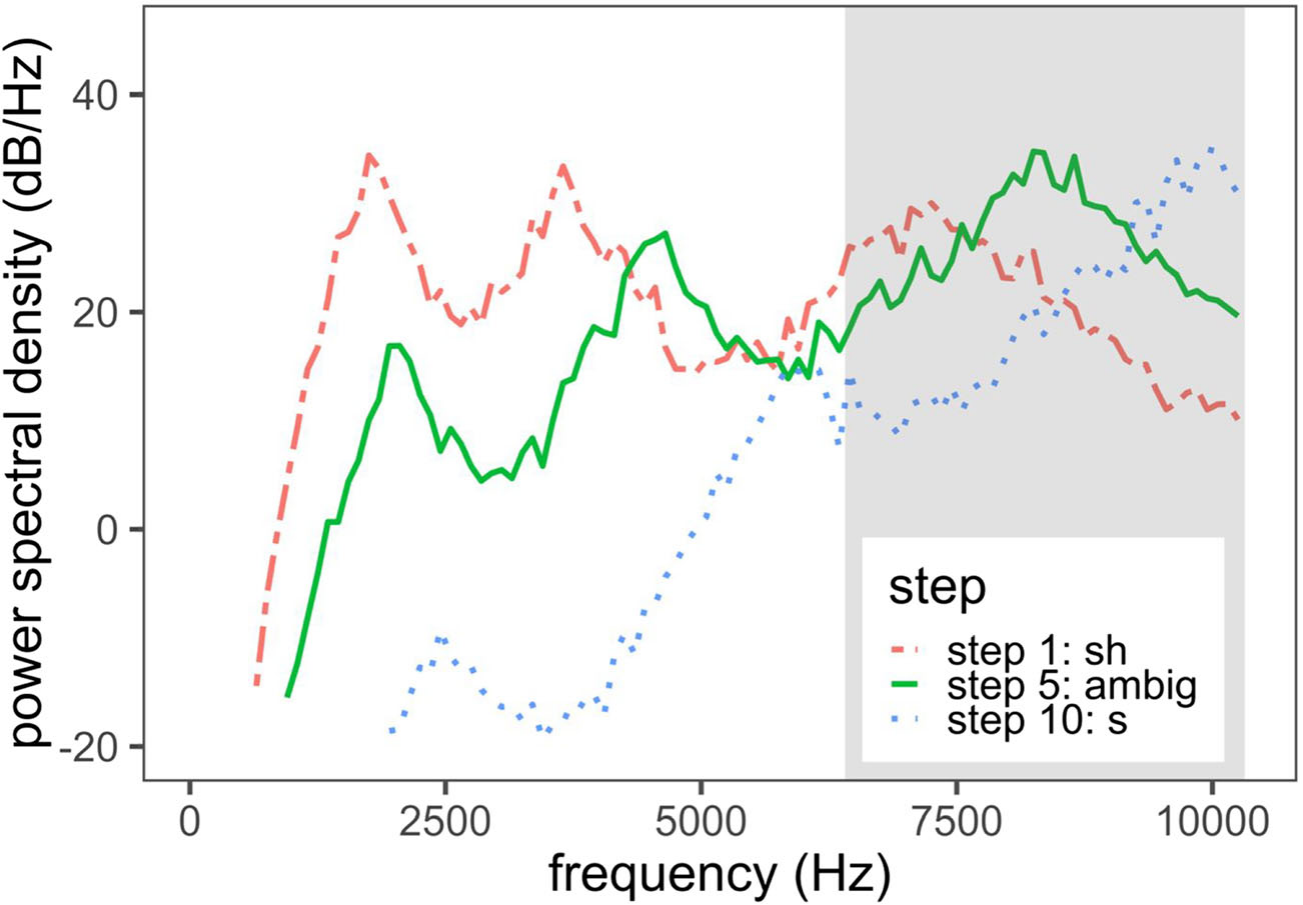
Long-term average spectra of the low endpoint (Step 1, most /ʃ/-like), middle point (Step 5, ambiguous), and high endpoint (Step 10, most /s/-like) of the /s/–/ʃ/ continuum in the present study. The shaded region ranges from 20 to 22 Bark (approximately 6.5 to 10.3 kHz) and highlights the frequency range most likely to be relevant for the /s/–/ʃ/ contrast with respect to the exposure stimuli

**Fig. 3:**
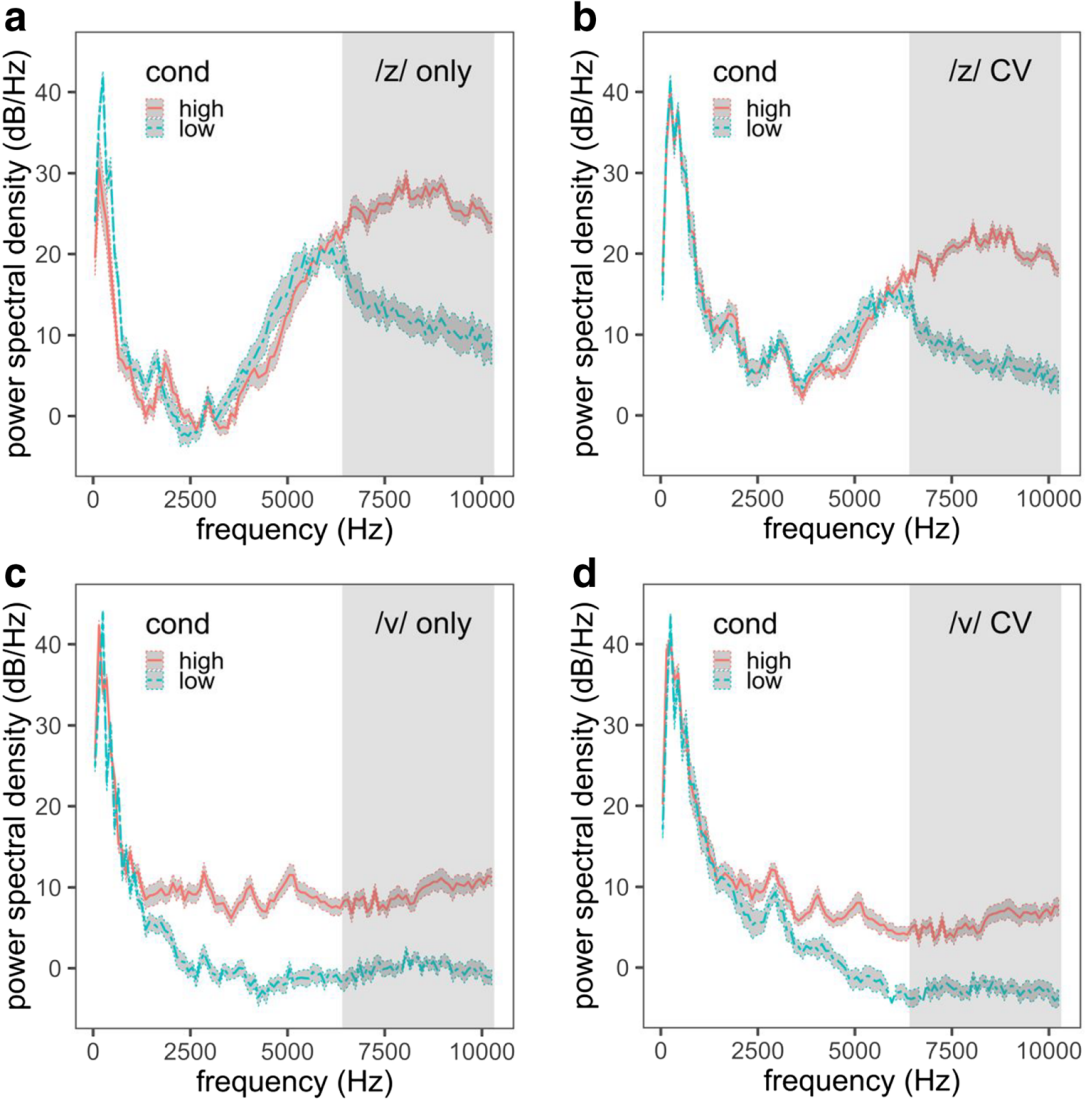
Long-term average spectra of (a) the high- and low-center-of-gravity (COG) exposure /z/s, (b) the high- and low-COG /z/s with the following vowel, (c) the high- and low-COG /v/s, and (d) the high- and low-COG /v/s with the following vowel. The rectangular shaded regions range from 20 to 22 Bark (approximately 6.5 to 10.3 kHz) and highlights the frequency range most likely to be relevant for the /s/–/ʃ/ contrast with respect to the exposure stimuli. The band around each contour reflects ± 1 standard error of the power spectral density across stimuli

Auditory contrast thus predicts the same pattern of transfer as the phonetic covariation account from /z/, but not from /v/, to /s/ and /ʃ/, though in a very different way. As is shown in Fig. 2, an important and relevant spectral difference between /s/ and /ʃ/ resides in the location of a mid- to high-frequency peak, which in the present study is located between approximately 20 and 22 Bark (or approximately 6.5 and 10.3 kHz; Traunmüller, 1990). Importantly, /z/ is also characterized by a mid- to high-frequency spectral peak within this same region (Fig. 3). Among the stimuli in the present study, the high-COG /z/s have a spectral peak between 21 and 22 Bark, whereas the low-COG /z/s have a spectral peak between 20 and 21 Bark. Any overlapping energy between the /z/ and /s/–/ʃ/ stimuli may result in a perceptual contrast effect. Specifically, the high-COG /z/ should give rise to perception of a /s/–/ʃ/ spectrum with a seemingly lower concentration of energy, resulting in a greater number of /ʃ/ responses. The low-COG /z/, in contrast, should give rise to perception of a /s/–/ʃ/ spectrum with a seemingly higher concentration of energy, resulting in a greater number of /s/ responses, especially relative to the high-COG /z/ exposure condition.^2^

Auditory contrast predicts little to no influence of /v/ exposure on /s/–/ʃ/ categorization, because /v/ is marked by relatively little energy in the relevant frequency range for sibilant categorization, regardless of its COG (Fig. 3). Because spectral overlap is minimal, contrast effects should be weak, if they are present at all. Importantly, nonspeech matched in LTAS (as well as duration and amplitude) to the /z/ stimuli should give rise to comparable effects on categorization.

### Present study

Motivated by the preceding discussion, in the present series of experiments, we examined short-term adaptation to /s/ and /ʃ/ based on various preceding auditory contexts: /z/-initial syllables, /v/-initial syllables, white noise matched in LTAS to the /z/-initial syllables, and alternating /z/-initial syllables and white noise. The primary aim was to elucidate the properties of the adaptation mechanism underlying rapid and generalized adaptation of speech perception. Specifically, do listeners generalize the properties of an auditory context to an ambiguous /s/–/ʃ/; if so, what types of auditory context trigger such generalization; and finally, what does this generalization reveal about the mechanisms underlying speech adaptation?

## Experiment 1: Exposure to /z/

The purpose of the first experiment was to determine whether exposure to the talker-specific phonetic realization of one fricative (specifically, /z/) can affect the categorization of members of a /s/–/ʃ/ continuum (see also Durvasula & Nelson, 2018). An influence of /z/ on the perception of /s/ and /ʃ/ would be expected under any of the three mechanisms discussed earlier—whether via active generalization from the talker-specific spectral properties of /z/ or via more general auditory-processing mechanisms. Two different speakers were presented in the experiment, one with a high-COG /z/ and one with a low-COG /z/, with speaker–condition pairings counterbalanced across participants (see also Kraljic & Samuel, 2006, for a related design). A positive result for this experiment would establish a baseline of generalized adaptation that would then set the stage for subsequent experiments aimed at distinguishing among the three mechanisms under consideration.

### Method

#### Participants

Twenty-eight participants (21 female, seven male) were recruited from the Johns Hopkins University undergraduate community. All were native speakers of American English. Twenty-three were monolingual, four were bilingual (with German, Hebrew, Korean, or Mandarin), and one was trilingual (with Cantonese and Mandarin). One participant reported having a speech impediment but no hearing impairment. In all the experiments presented here, participants were compensated with partial course credit.

#### Stimuli

##### Exposure stimuli: /z/-initial syllables

The exposure stimuli were /z/-initial consonant–vowel–consonant (CVC) syllables created by concatenating natural recordings. All recordings were selected from a corpus of CVC syllables from 22 native speakers of American English (15 female, seven male) recorded at New York University. The participants recorded CVC syllables in isolation as distractor items in an experiment on the perception and production of nonnative consonant clusters. In each trial, participants heard a prerecorded multisyllabic nonword and then saw a fricative-initial CVC syllable in standardized orthographic form, which the participant produced prior to producing the multisyllabic nonword. There were 12 unique presentation orders, and each CVC syllable was presented two or three times over the course of the experiment. The CVC syllables were composed by fully crossing the fricatives /*f* v ð θ s ʃ z ʒ/ with the vowels /i ɪ eɪ ɛ æ ʌ a ɔ oʊ ʊ u /, followed by /t/ (Jongman et al., 2000). Two /ʃ/-initial combinations were excluded, as they formed profane words. All recordings were made with a Zoom H4n digital recorder and an Audio-Technica ATM-75 head-mounted condenser microphone in a sound-attenuated booth at a sampling frequency of 44.1 kHz.

Four female speakers were selected from the corpus: one with a high-COG /z/, one with a low-COG /z/, and two with relatively neutral COGs in their realizations of /s/, /ʃ/, and /z/.^3^ The syllable rimes (i.e., the VC portion) of the exposure stimuli were selected from the two neutral-COG speakers, who were referred to as “Meg” and “Kim” in the experiment. For each of these two speakers and each of the ten vowels in the corpus, we selected a /z/-initial CVC syllable with a /z/ COG that was relatively medial in their productions. The VC portion of the syllable was extracted at zero-crossings (/z/ excluded) and normalized to 65 dB. Each stimulus included a final /t/, which was composed of a silent closure period and a short burst.^4^

The critical manipulation was the COG of the /z/. For each vowel, the highest-COG /z/ that could naturally be appended to the VC portion was selected from the speaker with overall high /z/ COGs. The same process was carried out for the female speaker with relatively low COGs across sibilants, but using the lowest-COG /z/ with natural concatenation. The mean COG of the high /z/ was 8021 Hz, with a standard deviation of 481 Hz, and the mean COG of the low /z/ was 6038 Hz, with a standard deviation of 731 Hz.

The /z/ durations were reduced to 85 ms, which was approximately the longest shared duration among the original productions. Original durations ranged from 99 to 164 ms for the high-COG speaker and 88 to 148 ms for the low-COG speaker. Throughout this procedure, consideration was taken to ensure an amplitude trajectory continuous with the beginning of the VC portion. All cuts were made at zero-crossings in the waveform, and the amplitude of each /z/ was normalized to 65 dB.

The high- and low-COG /z/s were concatenated with the vowel-matched VC portions from both Meg and Kim. Each stimulus was tapered at the beginning over a period of 50 ms (targeting the /z/ portion) to create a rising amplitude characteristic of the fricative. Then, 20 ms of silence was added to both ends of the stimulus. Altogether, there were four sets of ten /z/-initial stimuli: high /z/–Meg, low /z/–Meg, high /z/–Kim, and low /z/–Kim. The LTASs of the high- and low-COG /z/s, both alone and in the full syllables (CV portion), are shown in Fig. 3.

##### Categorization stimuli: /s/–/ʃ/ continua

An 11-point continuum was synthesized using a Bark-scale interpolation between the endpoints corresponding to /s/ and /ʃ/ (Winn, 2014). The endpoints were generated from white noise with specifications for three spectral peak locations, their slopes, and their relative amplitudes. The three peaks of the /s/ endpoint were located at 2.5, 6, and 10 kHz, with respective peak slopes of 25, 55, and 55 dB/octave. The relative amplitude of the first to the second peak was – 25 dB, and that from the second to the third peak, – 20 dB. For the /ʃ/ endpoint, the peaks were at 1.7, 3.5, and 7 kHz, with respective peak slopes of 35, 45, and 40 dB/octave. The relative amplitude of the first to the second peak was 5 dB, and that from the second to the third peak was 4 dB. The peak values for each endpoint were estimated from natural productions in the female subset of the CVC corpus and were also based on the authors’ assessment of relatively natural female /s/ or /ʃ/ production. All durations were 150 ms, with a rise time of 110 ms and a fall time of 30 ms. The intensity of the /s/–/ʃ/ segment was then scaled to 65 dB. The highest COG endpoint was excluded, resulting in ten steps in the continuum. Figure 2 shows the spectral shapes of the first, middle, and final segments of the continuum.

The members of the /s/–/ʃ/ continuum were then appended to the VC syllable rimes produced by Meg and Kim, to create a *seat*–*sheet* continuum (/i/ continuum) and a *suit*–*shoot* continuum (/u/ continuum). The VC tokens were chosen primarily on the basis of fluency and naturalness, as well as for having a relatively neutral fricative COG. For Meg, the VC portions came from recordings of *seat* and *shoot*, and for Kim, the VC portions came from recordings of *sheet* and *suit*. The VC portion was extracted at zero-crossings and was scaled to 65 dB. The /s/–/ʃ/ segments were appended to the onset, and 20 ms of silence was added to each end of the stimulus.

#### Procedure

Each participant received exposure to both COG levels in the experiment. Because a primary goal of the experiment was to examine talker-specific adaptation, COG level was crossed with speaker voice. COG order and speaker order were counterbalanced across participants resulting in four combinations (high /z/–Meg, low /z/–Kim; low /z/–Meg, high /z/–Kim; high /z/–Kim, low /z/–Meg; low /z/–Kim, high /z/–Meg). Each participant received both the high and low COG levels, but COG level was crossed with speaker voice such that a true within-speaker comparison was not possible. Although a baseline condition involving /s/–/ʃ/ categorization without preceding exposure stimuli would have helped in interpreting the direction of shifts, it might also have obscured the effects of generalized talker-specific adaptation by allowing participants to adapt to the talker’s range of /s/–/ʃ/ productions prior to the experimental manipulation. Care was taken to ensure that comparison of the aggregate /s/–/ʃ/ response curves as a function of COG level was interpreted merely as a difference between two participant groups and not as a shift in an individual’s response curve, either from the opposing COG level or from a baseline response curve.

Each trial consisted of an exposure sequence followed by categorization of a single member of an /s/–/ʃ/ continuum. In the exposure sequence, a /z/-initial syllable was presented twice. The speaker’s name and the intended (non)word were presented simultaneously on the screen (e.g., “Listen to Meg say the word ZATE . . .”). Listeners were then asked to categorize the initial fricative of a single syllable from one of the /s/–/ʃ/ continua in a two-alternative forced choice task. The response options were “S” and “SH.” There were 1.5 s of silence between the two /z/-initial stimuli, 1 s between the second /z/-initial stimulus and the /s/–/ʃ/ test stimulus, and 1.5 s between trials. Participants were presented six blocks of 20 trials for each speaker/exposure condition. Within a block, the 10 /z/-initial syllables were presented in random order twice, and each of the 20 /s/–/ʃ/ continuum members was presented once.

The first trial served as practice, in which the experimenter guided the participant through the structure of the exposure and categorization phases. Listeners were informed that the exposure words would always begin with /z/ and that some of the words would be familiar and others would be novel. Additionally, they were instructed to listen closely and to get to know the speaker’s voice.

### Results

Responses were analyzed with a Bayesian logistic mixed effects regression model implemented in the brms package for R (Bürkner, 2017). This package provides an interface to the Stan programming language, which can estimate complex model parameters with relative ease and speed by using a variation on a Hamiltonian Monte Carlo algorithm, the No-U-Turn sampler (Hoffman & Gelman, 2014). The Bayesian analysis returns a joint posterior distribution of the model parameters, and summary statistics are provided for each estimated marginal distribution. We report the estimated mean and 95% credible interval (CI) of the marginal posterior distribution for each effect under consideration. To evaluate whether an effect has had a meaningful influence on the outcome of the dependent variable (here, the /s/–/ʃ/ response), we consider the width of the 95% CI and, critically, whether that interval encompasses zero, which would indicate variation in the estimated direction of the effect. We refer readers to Bürkner for a thorough overview of the package, and Vasishth, Nicenboim, Beckman, Li, and Kong (2018) for a comprehensive tutorial on brms using phonetic data.

The Bayesian logistic mixed effects model predicted the binary /s/–/ʃ/ response (/s/ response = 1, /ʃ/ response = 0) from the condition (high = 0.5, low = – 0.5), vowel in the /s/–/ʃ/ continuum (/u/ = 0.5, /i/ = – 0.5), continuum step, speaker (Meg = 0.5, Kim = – 0.5), and the interactions between condition and vowel and between condition and speaker. The random effects structure included an intercept for the exposure word, an intercept for participant, and by-participants slopes for condition, vowel, continuum step, and the interaction between condition and vowel. Continuum step was converted to a numeric predictor scaled to have a mean of zero and a standard deviation of one. We employed the default prior distribution, which was an improper uniform distribution over real numbers. The parameter that controls step size, *adapt_delta*, was set to .999, to decrease the number of divergent transitions in sampling.

**Fig. 4:**
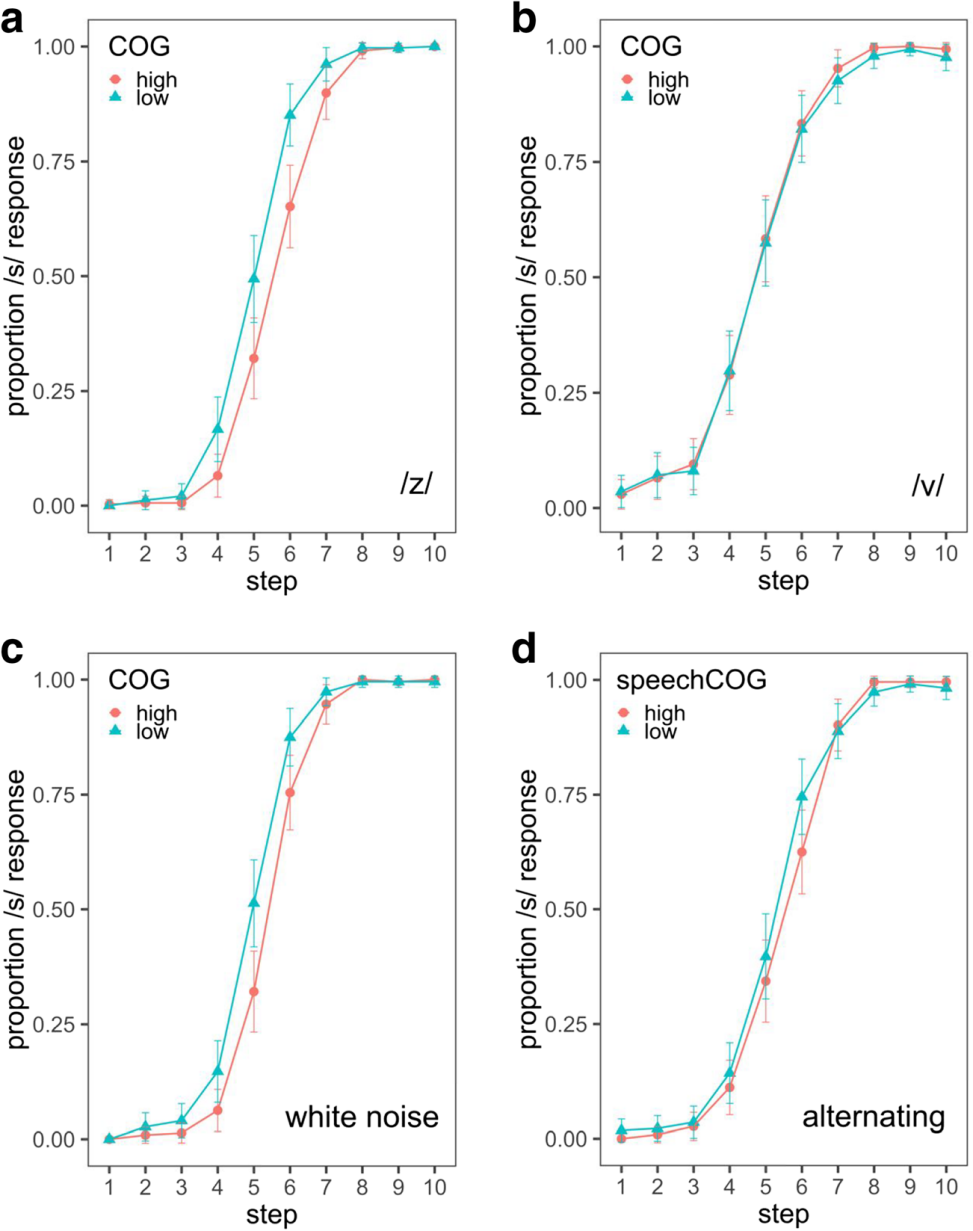
Proportions of /s/ responses following exposure to (a) the high- and low-center-of-gravity (COG) /z/ stimuli, (b) the high- and low-COG /v/ stimuli, (c) the long-term average spectrum (LTAS)-matched white noise, and (d) alternating speech and white noise with opposing COG levels

Substantially fewer /s/ responses were observed after exposure to a high-COG /z/ relative to a low-COG /z/ (β = – 1.62, 95% CI: [– 2.41, – 0.84]; Fig. 4a). The 95% CI around the estimate of this effect did not encompass zero, providing compelling evidence for the direction and strength of this effect. Consistent with previous perceptual findings, the following vowel also substantially influenced categorization, with an /s/ response being less likely in the context of /i/ than of /u/ (β = 2.54, 95% CI: [1.78, 3.29]; Mann & Repp, 1980). As expected, step number in the COG continuum also contributed to the response pattern, with higher steps being more likely to be called /s/ (β = 8.68, 95% CI: [7.24, 10.38]). The effect of speaker and the interactions between condition and speaker and between condition and vowel all had 95% CIs that spanned zero, suggesting that these factors had minimal or no influence on categorization (see the Appendix, Table 1). An additional model using data from the first block alone demonstrated evidence for substantially different rates of /s/ categorization based on the COG level after only minimal exposure (β = – 1.96, 95% CI: [– 3.28, – 0.91]).^5^

### Discussion

The results indicated that listeners transferred spectral properties from /z/ to /s/, and that generalization occurred early during exposure. These findings are consistent with all three presented accounts. The response patterns clearly differed between the high-COG and low-COG exposure groups, indicating a significant influence of COG exposure on /s/–/ʃ/ perception in at least one exposure group. Consistent with cue calibration, listeners could have inferred the talker-specific COG offset from exposure to /z/, and accordingly adjusted their expectations for /s/ and /ʃ/. Alternatively, listeners could have employed perceptual knowledge of the high empirical covariation of COG between /s/ and /z/ in order to adjust expectations about /s/ after exposure to /z/ (cue covariation). For example, a talker with a high-COG /z/ should also have a high-COG /s/, which would lead to an overall higher /s/–/ʃ/ boundary (and thus to fewer /s/ responses) than would a talker with a low-COG /z/. These findings are also compatible with auditory contrast: The high frequency peak in the high-COG /z/s might have dampened the perception of energy in following /s/–/ʃ/ stimuli, resulting in relatively fewer /s/ responses. Although additional manipulations were necessary to distinguish between the three adaptation accounts, these findings reveal that adaptation transfers across sibilant categories.

## Experiment 2: Exposure to /v/

In Experiment 2, we examined the differing predictions of the cue calibration account in comparison to the cue covariation and auditory contrast accounts. In particular, if listeners track talker-specific distributional information at the level of fricative cues, exposure to any one fricative category should affect perception of any other fricative category by altering the aggregate mean of that cue over all fricatives. However, the mean COG of /v/ does not covary with the mean COG of /s/ or /ʃ/ across talkers. If listeners exploit perceptual knowledge of phonetic covariation, then listeners should not generalize the talker-specific COG from /v/ to /s/, or they should do so less than we found after exposure to /z/. A lack of generalization would also be consistent with auditory contrast, as the spectrum of /v/ contains relatively little mid- to high-frequency energy (i.e., the range relevant for /s/–/ʃ/ categorization, as shown in Fig. 3). The present experiment followed the same design as the preceding one, but we replaced the high- and low-COG /z/ exposures with exposures to high- and low-COG /v/.

### Method

#### Participants

Experiment 2 was completed by 28 participants (15 male, 13 female) from the Johns Hopkins undergraduate community who had not participated in Experiment 1. Twenty-seven of the participants were native speakers of American English, and one participant was a native speaker of Mandarin but was fully fluent in English. Of the speakers, 25 were monolingual, and three were bilingual with English (Korean or Mandarin).

#### Stimuli

##### Exposure stimuli: /v/-initial syllables

The /v/-initial stimuli were created using approximately the same procedure as for the /z/-initial stimuli. Each stimulus was composed of a high- or low-COG /v/ concatenated with a VC syllable rime from the two neutral COG speakers (Meg and Kim). All recordings were selected from the corpus of fricative-initial CVC syllables described in the Experiment 1 Stimuli section. For the /v/ portion of the stimulus, two female speakers, one with a relatively high-COG /v/ and one with a relatively low-COG /v/, were identified and their recordings of /v/ extracted. For the VC portion of the stimulus, recordings from the same two neutral-COG speakers (Meg and Kim) were used, which allowed the /s/–/ʃ/ continua from Experiment 1 to be used as the speech targets. As before, the syllable rimes were extracted from /v/-initial words with unique VC portions for each speaker, each with a different vowel (/i ɪ eɪ ɛ æ ∧ a ɔ oʊ u/). All splices were made at zero-crossings, and the amplitude was normalized to 65 dB.

A high- and a low-COG /v/ was chosen for each vowel type. We aimed to select a /v/ that preceded the same vowel type of the syllable being created in the original recording. In cases when this was not possible, we used a neighboring vowel in the F1×F2 vowel space. Each /v/ was truncated to 85 ms, and the intensity was ramped over the first 30 ms, for a more natural sound. The amplitude was then normalized to 65 dB. The high- and low-COG /v/s were then concatenated with the vowel-matched VC portions from Meg and Kim, and 20 ms of silence was appended to both ends. The mean COG of the high /v/ was 4655 Hz with a standard deviation of 1200 Hz, and the mean COG of the low /v/ was 2119 Hz with a standard deviation of 844 Hz. The LTAS of the high- and low-COG /v/s and full syllables (CV portion) are shown in Fig. 3.

##### Categorization stimuli

The stimuli presented for categorization were members of the same /s/–/ʃ/ continua used in Experiment 1 (Stimuli section).

#### Procedure

Experiment 2 followed the same procedure as Experiment 1, except that the /z/-initial syllables were replaced with /v/-initial syllables (see the Exp. 1 Procedure section).

### Results

#### Exposure to /v/

The categorization responses were again submitted to a Bayesian logistic mixed effects model with the same structure as in the analysis of Experiment 1 (Results section). The model predicted the probability of /s/ response from the condition, vowel, speaker, and continuum step, as well as the interactions between condition and vowel, as well as condition and speaker. The model also included a random intercept and slopes for condition, vowel, continuum step, and the interaction between condition and vowel for participant, as well as a random intercept for the exposure word.

The effects of /v/ COG on the /s/–/ʃ/ response did not differ substantially between exposure groups: The estimated effect and CI were centered near zero (β = 0.04, 95% CI: [– 0.48, 0.55]; Fig. 4b). Categorization was substantially influenced by the following vowel (/i/ or /u/, β = 1.50, 95% CI: [0.57, 2.43]) and continuum step (β = 6.33, 95% CI: [5.32, 7.43]). The 95% CIs around the estimated influences of speaker, the interaction between condition and vowel, and the interaction between condition and speaker all contained zero, indicating minimal influence on categorization (see the Appendix, Table 2).^6^

### Discussion

Contrary to the predictions of the cue calibration account, listeners did not generalize talker-specific spectral properties from /v/ to the /s/–/ʃ/ contrast, indicating that fricatives are not treated equally with respect to their spectral properties in adaptation to a novel talker. These findings are nevertheless consistent with both cue covariation and auditory contrast. Listeners could exploit phonetic knowledge that /s/ and /v/ are statistically independent across talkers in a way that /s/ and /z/ clearly are not. The short-term adaptation effects could also be accounted for by the spectral energy distribution: the perception of /s/ and /ʃ/ may be unaffected by exposure to high or low variants of the labiodental fricative, as /v/ contains very little mid- to high-frequency energy. The statistical relationship between the exposure items in Experiments 1 and 2 (/z/ and /v/) will be assessed in combination with those from Experiment 3 (speech-shaped noise) in the following section.

Without a baseline condition excluding exposure to the speaker’s /v/, we cannot conclusively state whether or not listeners shifted their response curve in response to the stimuli. If indeed listeners adapted to the stimuli at hand, this effect would have to be driven by the vocalic portion of the stimuli, since otherwise there was no difference between the high- and low-COG response curves. Although adaptation to /s/ and /ʃ/ based on a speaker’s vowel may be suggestive of some phonetic relationship between a speaker’s vowels and their sibilants, it would not necessarily discriminate between the proposed adaptation mechanisms at hand. The findings do, however, strongly indicate that listeners did not substantially adapt to a speaker’s /s/ and /ʃ/ on the basis of the spectral realization of /v/.

## Experiment 3: Exposure to speech-shaped noise

In the preceding experiments we examined adaptation to linguistic auditory stimuli, and the results were consistent with both the cue covariation and auditory contrast adaptation accounts. These accounts diverge, however, with respect to nonlinguistic auditory stimuli. In particular, auditory contrast makes the additional prediction that appropriately constructed nonlinguistic exposure items should yield the same pattern of adaptation as linguistic exposure items. We tested the predictions of this using the same design and procedure as Experiment 1, but with white noise matched in LTAS, duration, and amplitude to the /z/-initial syllables.

### Method

#### Participants

An additional 28 participants (16 female, 12 male) from the Johns Hopkins undergraduate community completed Experiment 3. Twenty-seven of the participants were native speakers of American English, and one participant was a native speaker of Mandarin but spoke American English fluently. Including the native Mandarin speaker, two participants were bilingual with English (Mandarin, Cantonese). Participants received partial course credit for completion of the experiment.

#### Stimuli

##### Exposure stimuli: Noise

White noise was matched in LTAS, duration, and amplitude of the CV portion of each /z/-initial stimulus using Praat (Winn, 2014). The noise signal was tapered at each end over a period of 50 ms and then matched in amplitude to the CV portion of the corresponding speech syllable. The final /t/ closure and burst in the /z/-initial stimuli were replaced with a silent interval matched in duration.^7^ Finally, 20 ms of silence was appended to each end of the signal. The resemblance between the frication noise and white noise was intentional, so that the stimuli would also largely be matched on signal type (e.g., turbulence and aperiodicity). The high noise stimuli had a mean COG of 5280 Hz, with a standard deviation of 2065 Hz (cf. 8021 Hz with a standard deviation of 481 Hz for the high-COG /z/ portion of the speech stimuli), and the low noise stimuli had a mean COG of 2400 Hz, with a standard deviation of 1207 Hz (cf. 6038 Hz with a standard deviation of 731 Hz for the low-COG /z/ portion of the speech stimuli). The apparently lower COG reported for the noise stimuli reflects the fact that each stimulus was matched in LTAS to the full CV portion of the /z/-initial stimuli. The white noise resembled radio static, and all but one listener reported that it was not perceived as speech. (That one listener likened the noise to a very sustained voiceless velar or uvular fricative, which is not native to English.) The relevance of the similarity between the source properties between fricatives and white noise is discussed further in the General Discussion.

##### Categorization stimuli

In this experiment we used the same /s/–/ʃ/ categorization stimuli as in the previous two experiments (see the Exp. 1 Stimuli section).

#### Procedure

The procedure in Experiment 3 followed that of Experiment 1, except that the /z/-initial exposure stimuli were replaced with the matched white noise stimuli. In contrast to Experiment 1, there were four blocks of 20 trials, as opposed to six blocks. Given the early presence of the effect in Experiment 1 and the fact that participants would be listening to noise, we judged that a shorter experiment would be effective and preferable. The first trial again served as practice, in which the experimenter guided the participant through the structure of the exposure and categorization phases. Listeners were told they would be listening to a new speaker (either Meg or Kim), but that they would first hear two identical nonspeech sounds. Listeners were instructed to listen closely to the exposure stimuli and to the speaker’s voice.

### Results

#### Exposure to speech-shaped noise

The proportions of /s/ responses following exposure to the high- and low-COG white noise stimuli are shown in Fig. 4c. A logistic mixed effects model with the same structure as in Experiments 1 and 2 was used to assess the results. Paralleling the corresponding model in Experiment 1 (/z/ exposure), COG condition, continuum vowel, and continuum step substantially influenced the rate of /s/ categorization (condition, β = – 1.41, 95% CI: [– 2.17, – 0.68]; vowel, β = 2.69, 95% CI: [1.88, 3.53]; step, β = 9.02, 95% CI: [7.57, 10.76]). The effect of speaker, which corresponds here to a set of exposure stimuli along with the voice present in the /s/–/ʃ/ continuum, had a marginal influence on categorization (β = 0.72, 95% CI: [0.03, 1.41]). The interactions between condition and vowel and between condition and speaker did not reliably influence categorization (see the Appendix, Table 3). The effect of condition was already present within the first block of exposure (β = – 1.63, 95% CI: [– 2.82, 0.62]).^8^

#### Comparison between exposures to /z/, /v/, and speech-shaped noise

A statistical assessment of the differences between the exposure types was conducted in a combined Bayesian logistic mixed effects model with data from all three experiments. The model included fixed effects of experiment (/z/, /v/, or noise exposure), COG condition, vowel, step, and speaker, as well as the full interactions between experiment, condition, and vowel. The model also included a random intercept and slopes for condition, vowel, step, and the interaction between condition and vowel, for participants, along with a random intercept for the exposure word rime. The binary categorical factors were sum-coded as before, and the three-level categorical factor of experiment was treatment-coded, with /z/ as the baseline level.

The patterns of results in the /z/ and /v/ exposure experiments were qualitatively different from one other, whereas those in the /z/ and speech-shaped noise exposure experiments were qualitatively similar. The logistic mixed effects model revealed a main effect of COG condition (β = – 1.54, 95% CI: [– 2.15, – 0.93]); however, this effect was modulated by experiment. In particular, listeners with exposure to the high-COG condition in the /v/ experiment were more likely to respond /s/ than were listeners in the high-COG condition in the /z/ experiment, which is the effect predicted, given adaptation to a high-COG /z/ (Condition × Experiment_/v/–/z/_: β = 2.99, 95% CI: [1.26, 4.79]). The effect of COG condition, however, did not differ between the /z/ and speech-shaped noise exposure experiments (Condition × Experiment_noise–/z/_: β = 0.52, 95% CI: [– 1.19, 2.29]). As expected, main effects of continuum step and continuum vowel influenced categorization in all three experiments (step, β = 7.93, 95% CI: [7.22, 8.67]; vowel, β = 2.40, 95% CI: [1.65, 3.17]). Apart from the difference between the /z/ and /v/ experiments in the influence of COG condition on /s/–/ʃ/ categorization, no additional meaningful differences were observed among the /z/, /v/, and speech-shaped noise experiments (see the Appendix, Table 4).

### Discussion

The LTAS-matched white noise stimuli had an influential effect on /s/–/ʃ/ categorization that was statistically indistinguishable from the effect of /z/-initial stimuli, with respect to the difference between high- and low-COG stimuli. Although the direction and magnitude of the shifts for the /z/ and noise exposure experiments may have differed, the difference between the high- and low-COG conditions for both groups was highly comparable.^9^ This pattern thus provides strong evidence for auditory contrast, in that both linguistic and nonlinguistic exposure stimuli with energy in the relevant frequency range for categorization had comparable effects on the perception of coronal fricatives. The auditory contrast account is also consistent with the results of the /v/ exposure experiment, given that the spectra of coronal and labial fricatives overlap minimally and therefore should not interact contrastively. Because the difference in the response curves observed in the present experiment is uniquely predicted by the auditory contrast account (within the set of alternative accounts that we consider), at least on parsimony grounds alone, the parity between /z/-initial syllables and noise-matched adaptors casts doubt on the cue covariation account for this type of perceptual generalization.

Adaptation by auditory contrast cleanly accounts for these local adaptation results: The preceding acoustic context, regardless of its linguistic status, affects perception of the following speech sound, provided that there is sufficient energy in the relevant frequency range. Nevertheless, evidence from long-term talker adaptation and talker familiarity effects suggests that listeners may also be sensitive to talker-specific linguistic realizations (e.g., Eisner & McQueen, 2006; Kraljic & Samuel, 2005; Nygaard & Pisoni, 1998). Listeners could merely associate general auditory properties with a talker’s voice, although these long-term effects do implicate sensitivity to the relationship between a phonetic (linguistic) realization of a speech sound and a particular talker (e.g., talker-specific linguistic representations). If, indeed, perceptual learning of phonetic properties occurs, this would indicate an ability to disassociate linguistic from nonlinguistic evidence regarding a talker’s voice. In Experiment 4, we explicitly addressed whether linguistic information can be distinguished from nonlinguistic information in local contexts, and specifically, whether listeners make preferential use of linguistic information in a context with alternating speech and speech-shaped noise.

## Experiment 4: Exposure to alternating /z/ and speech-shaped noise

Reliable influences of linguistic and nonlinguistic exposure on fricative categorization were found in Experiments 1 and 3 after exposure to /z/-initial and noise-matched stimuli. The goal of the present experiment was to determine the relative weighting of these two types of stimuli. The experiment had a structure similar to that of the preceding experiments, in which exposure alternated with /s/–/ʃ/ categorization within each trial. However, in this case, each exposure consisted of alternating /z/-initial syllables and speech-shaped noise with opposite COG levels.

### Method

#### Participants

Twenty-eight participants (15 male, 13 female) from the Johns Hopkins undergraduate community completed Experiment 4. Twenty-six of the participants were native speakers of American English, one participant was a native speaker of Korean, and one a native speaker of Cantonese, but both of the latter participants grew up also speaking English. An additional five participants completed the experiment but were not included in the analysis, due to experimenter error. Nineteen of the participants were monolingual, seven were bilingual (with Arabic, Korean, Mandarin, or Portuguese), and two were trilingual (with Cantonese and Mandarin or with Hindi and Tamil). All participants received partial course credit for participation.

#### Stimuli

##### Exposure stimuli

The /z/-initial syllables and LTAS-matched noise stimuli described in the Stimuli sections from Experiments 1 and 3 were concatenated to form a sequence of speech and noise that alternated in the direction of the COG manipulation. For each speaker, the high-COG /z/ stimuli were paired with the low-COG noise stimuli, and the low-COG /z/ stimuli were paired with the high-COG noise stimuli. The sequence of speech and noise was repeated once, for a total of four presentations (i.e., the entire sequence at the beginning of a trial was speech–noise–speech–noise).

##### Categorization stimuli

The /s/–/ʃ/ continua were the same as those in the previous experiments (see the Exp. 1 Stimuli section).

#### Procedure

Because two sets of speech and noise stimuli differing only in COG level were created for each speaker, the high- and low-COG manipulations for the speech and noise stimuli could be fully crossed. Listeners received the high- or low-COG /z/ stimulus interleaved with the opposite COG-level noise stimulus for the first speaker; for the second speaker, the COG levels for the speech and noise were switched. Speaker order and exposure order were counterbalanced across participants.

Within each trial, exposure comprised two alternations of the /z/-initial syllable and the noise stimulus. The pairing of the /z/-initial syllable and the noise stimulus was constant: The noise stimulus corresponded to the /z/-initial syllable with the same VC portion, but had the opposite /z/ COG level. The speaker’s name and the intended (non)word were presented on the screen during the audio presentation (e.g., “Listen to Meg say the word ZATE, followed by a brief sound . . .”). Immediately following exposure, participants categorized the initial fricative of a randomly selected /s/–/ʃ/ test stimulus. There were 500 ms of silence between members of the sequence. This amounted to 1.5 s of silence between exposure stimuli within a trial, which, in total, was the same amount of silence between exposure items as in the preceding experiments. An additional 1 s of silence preceded the onset of the /s/–/ʃ/ test stimulus, and 1.5 s of silence separated trials. There were also 20 ms of silence flanking the two audio files within each trial (the sequence of exposure stimuli and the test stimulus). The experiment consisted of four blocks of 20 trials each. Each unique sequence was presented twice within a block, and the 20 /s/–/ʃ/ test stimuli were presented once. The exposure and test stimulus pairing was randomized for each round of the four conditions (two speakers, two orders of COG level).

To adjust participants to the alternation of speech and noise, participants were again guided through the initial trial by the experimenter. Because this experiment involved two alternating modalities, the initial trial was also preceded by two practice exposure trials with speech and LTAS-matched noise generated from an unrelated voice. The words *bird* and *pink* were selected from the American Spoken Lexicon Corpus (Seidl-Friedman, Kobayashi, & Cieri, 1999) for their relatively high lexical frequency, for being similarity to the exposure stimuli in their monosyllabicity, and because they did not contain any fricative consonants or word-initial coronals. The corresponding noise stimulus was matched in LTAS, duration, and amplitude to each syllable, excluding the final stop consonant. Following the practice trials, listeners were given the same instructions as in the preceding /z/ and noise exposure experiments.

### Results

The results were analyzed in the same way as those of the previous experiments, with a Bayesian logistic mixed effects model predicting the probability of /s/ responses. The effect of condition trended in a direction consistent with preferential weighting of speech, in that listeners were less likely to respond /s/ following a high-COG /z/ (and low-COG noise). However, the 95% CI contained zero, indicating that the direction and strength of the effect was not reliably observed (β = – 0.48, 95% CI: [– 1.10, 0.13]; Fig. 4d). This suggests that the opposing influences of the speech and noise COGs largely canceled each other out. The model also revealed reliable influences of the vowel (β = 2.11, 95% CI: [1.50, 2.79]) and continuum step (β = 7.09, 95% CI: [5.86, 8.48]). The main effect of speaker and the interactions between condition and vowel and between condition and speaker had wide uncertainty, with means around zero (see the Appendix, Table 5).^10^

### Discussion

The null effect of the speech COG condition strongly suggests that the opposing linguistic and nonlinguistic exposure stimuli had inverse effects on categorization. Though adaptation to the linguistic stimuli was numerically greater than adaptation to the nonlinguistic stimuli, the statistical analysis indicated that this difference was not reliable, suggesting equivalence in the strength of adaptation to the linguistic and nonlinguistic stimuli over a short timescale. We would have expected a significantly stronger influence of linguistic than of nonlinguistic stimuli on speech adaptation if a mechanism based on cue covariation had overridden the auditory contrast.

One alternative account of the findings may be that the preceding stimuli had no effect on the categorization stimulus. In contrast to the previous experiments, in the present experiment we used an interval of 500 ms between exposure stimuli. However, in all four experiments, the total silent duration between exposure items within a trial amounted to 1.5 s, and the interval between the final exposure stimulus and onset of the categorization stimulus was always 1 s. The spectral contrast effect may be modulated or entirely altered, depending on whether the preceding stimuli were separated by long or short silent intervals and by the frequency of their repetition (see Holt, 2005). Though this might be true, we find it unlikely that the preceding stimuli would have no influence on perception of the test stimulus, especially as the interval preceding the test stimulus was the same across experiments. Future research should nevertheless examine the influence of the silent-interval duration and its frequency on spectral contrast effects in perception.

## General discussion

The present findings revealed generalized perceptual adaptation of an /s/–/ʃ/ contrast from spectral properties in /z/-initial syllables and LTAS-matched white noise, but not /v/-initial syllables. Furthermore, no significant preference was given to speech stimuli over nonspeech stimuli, as demonstrated by the lack of generalization to the /s/–/ʃ/ contrast from contrasting /z/-initial syllables and white noise. These findings improve our understanding of adaptation mechanisms involved in speech perception, particularly over relatively short timescales. The cue calibration account incorrectly assigned equal relevance to all segments with a shared cue, and although the cue covariation account made accurate predictions regarding adaptation from speech contexts, neither the cue calibration nor the cue covariation account could account for adaptation from nonspeech. Auditory contrast, in comparison, adequately accounted for the patterns of adaptation from both speech and nonspeech contexts.

Adaptation in speech perception occurs at varying timescales with varying mechanisms. The present study examined adaptation to the speech of a novel talker over a short timescale and revealed a mechanism of adaptation that was specific to particular auditory contexts, as opposed to speaker characteristics or linguistic status. In the following discussion, we first consider some of the assumptions involved in the framing of the mechanisms we considered here, and also how these mechanisms may differ from others. In the second section, we consider the relative weighting of linguistic and nonlinguistic influences in speech adaptation with respect to the spectral and temporal constitution of the input or precursor stimulus. Very generally, adaptation mechanisms may differ in their handling of linguistic and nonlinguistic input, as well as in the timescale at which the mechanisms become relevant. In the third section, we discuss the temporal window of integration and consider whether general auditory mechanisms may play a stronger role in perceptual adaptation at shorter timescales than would a linguistic adaptation mechanism.

### Framing of the present experiment

Talker-specific adaptation likely involves many mechanisms, including processes of intrinsic normalization, in which listeners exploit information internal to a given speech sound (e.g., Ainsworth, 1975; Strange, Verbrugge, Shankweiler, & Edman, 1976), distributional learning, in which listeners track talker-specific distributional properties of a given phonetic cue and category (e.g., Clayards, Tanenhaus, Aslin, & Jacobs, 2008; Kleinschmidt & Jaeger, 2015), modulation by top-down influences such as knowledge of talker gender or dialect (e.g., Kleinschmidt & Jaeger, 2015; Strand & Johnson, 1996), and extrinsic normalization, in which listeners exploit talker-specific information from multiple speech segments to refine expectations about an individual segment (e.g., Ainsworth, 1975; Assmann, Nearey, & Hogan, 1982; Nearey, 1978). Understanding speech adaptation requires proper definition of the proposed mechanisms and their interactions. The present study undertook a unique and principled approach to defining one adaptation mechanism that may underlie *rapid* and *generalized* adaptation in speech processing.

Our investigation of rapid and generalized adaptation, as opposed to long-term and direct adaptation (i.e., to one particular speech sound) resulted in particular decisions about stimulus presentation and measurement. Unlike in many other studies of fricative perception, there was temporal adjacency between the context and test stimuli. For that same reason, we also considered mechanisms involving adaptation to talker-specific linguistic properties, as well as those involving adaptation to the local auditory context. Defining these mechanisms and working out their predictions in turn required certain assumptions. For the linguistic mechanisms, we drew inspiration from previous phonetic research: as described in the Introduction, adaptation based on cue calibration relates to previous proposals involving rescaling or calibration of phonetic cues that are relevant to an entire class of speech sounds (e.g., McMurray & Jongman, 2011; Nearey, 1978). Adaptation using cue covariation derives from speech production findings demonstrating strong phonetic covariation among several, but not all speech sounds (e.g., Chodroff & Wilson, 2017). Both mechanisms require selecting phonetic cues, and one may reasonably wonder whether we selected the right ones for fricative perception. Though McMurray and Jongman included over 20 cues to fricative perception in their cue-based model of adaptation, they acknowledge these cues may not be the exact ones used in perception.

Although we cannot be certain that listeners track COG specifically, the findings revealed general sensitivity to aspects of the spectral shape. What does this mean for the cue calibration and cue covariation accounts? The set of speech sounds delimited as relevant for adaptation constitutes the primary difference between cue calibration and cue covariation. For the sake of argument, we could assume that the true cue is indeed COG (or spectral peak): in this article, we considered the full class of fricatives; however, COG could theoretically be measured from any and all speech sounds in a speaker’s inventory. The scope of a cue calibration account is therefore too broad. Some constraint must be in place to ensure that listeners match like with like when adapting to certain phonetic or auditory dimensions. In contrast, if the proper cue is not COG, but rather other aspects of the spectral shape including dynamic properties of the spectrum (e.g., Reidy, 2015, 2016), it is certainly the case that the class of sibilants have more spectral similarities to one another than to the larger class of fricatives (specifically, sibilants have a strong spectral peak and an overall high amplitude). Moreover, the aspects of the spectrum that are perceptually extracted must extend beyond phonetic-specific cues given that listeners adapted in a highly comparable manner to both linguistic and nonlinguistic exposure stimuli.

### Relative weighting of linguistic and nonlinguistic precursors

The similarity in adaptation between /z/-initial syllables and LTAS-matched white noise is consistent with some degree of parity between these exposure, or precursor, types, at least at this timescale. Certain adaptation mechanisms in speech perception may therefore be less sensitive to the linguistic status of the stimulus. Several studies examining a similar temporal domain of adaptation have, however, reported *asymmetrical* perceptual effects of speech and nonspeech precursors. For example, Sjerps, Mitterer, and McQueen (2011) observed stronger contrast effects from an original speech stimulus relative to its spectrally rotated counterpart, and Watkins and Makin (1996) reported a larger shift in listeners’ perception of members of an /æpt/–/ɑpt/ continuum following an F2 manipulation in speech relative to spectrally matched noise. Mitterer (2006) also found a significant effect of a speech stimulus with an F2-manipulation, but not LTAS-matched noise on the perception of following /e/ and /ø/. The preceding studies found a stronger effect of speech than nonspeech stimuli on perception, though at least one study has reported a stronger effect of nonspeech than speech stimuli on perception: Laing et al. (2012) found an overall stronger effect of a sine-wave tone series with an F3 manipulation on the perception of ambiguous /d/–/g/ sounds relative to the corresponding speech contexts of /l/ and /ɹ/. These findings give rise to the question of the source of such asymmetries between speech and nonspeech precursors.

Variation in adaptation may be accounted for by differences in the acoustic properties of the context stimuli, regardless of their linguistic status, and specifically, by the degree to which they excite frequency bands relevant for categorization. Signals of different types (e.g., periodic vs. aperiodic/turbulent) may also give rise to different adaptation effects. Furthermore, there is converging evidence for a relationship between the gain of the context stimulus (in relevant frequencies) and the strength the adaptation effect. Stilp, Anderson, and Winn (2015) examined contrast effects on members of an /ɪ/–/ɛ/ F1 continuum and established that the magnitude of the effect was modulated by the gain of the preceding F1 peak, but not by bandwidth. Relatedly, Stilp and Assgari (2017) found a linear relationship between filter gain and the magnitude of the spectral contrast effect on categorization of voiced stop consonants /d/ and /g/ following speech precursors with a relatively high or low F3 region. If contrast effects are most strongly predicted by the spectral structure of a signal, then temporally reversed speech should have a comparable spectral contrast effect as corresponding forward speech. Indeed, Watkins and Makin (1994) found that, whereas forward speech had a marginally stronger influence on the perception of a subsequent vowel, the effect of reversed speech was not significantly different.

Asymmetries in spectral contrast effects between speech and nonspeech precursors have also been observed when the nonspeech precursor is derived through a spectral rotation of the original speech stimulus. The asymmetry likely resulted from the change in the locus of energy within the frequency spectrum (see Sjerps et al., 2011). In addition, Laing et al. (2012) reported stronger adaptation to a /d/–/g/ contrast following a tone sequence than following /al/ and /ar/ syllables. The authors, however, noted that the tone sequence had a more peaky F3 region relative to the corresponding speech stimuli. This explanation aligns with the findings above regarding the relevance of frequency gain for spectral contrast effects.

An additional explanation for asymmetry in the influence of speech and nonspeech stimuli might have to do with the type of signal presented. Though only speculative, adaptation to vowels and adaptation to fricatives could depend on whether the signal is periodic or turbulent. In the present study, the context and categorization stimuli were primarily turbulent signals, and were thus relatively well-matched in signal type. In contrast, Watkins and Makin (1996) and Mitterer (2006) found mismatches in the effect of context on categorization depending on signal type: in both cases, noise stimuli matched in spectral properties to vowels resulted in substantially weaker contrast effects in the perception of a following vowel than corresponding vocalic precursors. Additional research would be necessary to examine the relevance of signal type for spectral contrast effects. One natural extension of the present experiment would be to manipulate tones in the critical frequency bands for the /s/–/ʃ/ contrast to determine whether these give rise to comparable degrees of adaptation as white noise stimuli.

Relatedly, although white noise has not been shown to affect the perception of vowels, it could still be categorized as *linguistic*, and therefore relevant in the perception of fricatives. Frication noise is highly comparable to modified white noise and can easily be generated in the vocal tract. Even the fricatives synthesized for the present experiment were derived from a white noise base. Perceiving speech-matched white noise as linguistic in form would suggest that listeners extract relevant cues from this stimulus for categorization and adaptation. Because all the precursor stimuli would be categorized as linguistic, an adaptation mechanism using cue covariation and one based on auditory contrast would be indistinguishable in their accounts of the present findings.

### Temporal window of integration

In addition to understanding the type and composition of stimuli that affect subsequent perception, it is also useful to flesh out the temporal window of auditory integration in perceptual adaptation to speech. There are two primary aspects of the problem to consider: first, which auditory stimuli are involved, and at which timescales, and second, which mechanisms are involved, and at which timescales. Because evidence from the present study pointed to a strong role of auditory contrast, we consider first how a general auditory effect like spectral contrast may be affected by the timing and temporal sequence of auditory stimuli. We then consider how additional mechanisms of adaptation may play a role at timescales longer than those tested in the present study.

Spectral contrast has been argued to exist at multiple timescales but all within relatively early stages of processing. These range from automatic effects that may occur in the peripheral auditory system to ones that may occur slightly later in central processing (e.g., Holt, 2005; Sjerps et al., 2011; Watkins, 1991). The adaptation observed in the present experiment occurred with an interval of at least 1 s between the relevant spectral manipulation and the onset of the /s/–/ʃ/ stimulus. The period between the relevant manipulation and the following stimulus is even longer when considering the temporal composition of the various precursor stimuli. Specifically, the influential spectral region in the speech stimuli was not sustained throughout the duration of the auditory presentation: The relevant spectral manipulation was present only in the /z/, which constituted the first 85 ms of the stimulus; the following vowel did not contain substantial energy above approximately 5000 Hz. Taking this into consideration, the full interval between the spectral manipulation in the /z/ and the following /s/–/ʃ/ stimulus was approximately 1.3 to 1.6 s in duration. Relatedly, in the experiment with alternating speech and nonspeech exposure stimuli, the properties of the exposure stimulus that immediately preceded the /s/–/ʃ/ categorization did not overwhelm the opposing influence of the preceding exposure stimuli. Instead, listeners appeared to integrate over all preceding auditory stimuli within the sequence. This integration period was at least 1.3 s in duration and contained intervals of both silence and sound. This finding parallels Holt (2005), in which contrast effects could be observed with up to 1.3 s of just silence intervening between the precursor and target speech sound, and a full integration period of almost 3.5 s over an entire sequence of tones in either a high or low F3 region; the tone immediately preceding the test stimulus could not account for the adaptation effect.

The relatively short period between precursor and test stimuli raises the question as to how aspects of memory, and in particular, auditory sensory memory, may have influenced adaptation effects. Auditory sensory memory (ASM) is characterized as a type of echoic memory, in which auditory stimuli are retained in memory at high-resolution for a short period of approximately three to five seconds (Nees, 2016; Neisser, 1967). Indeed, the precursor and test stimuli occurred within the implicated 3-s to 5-s interval, suggesting that the precursor stimulus would have been retained in memory as a high-fidelity representation. After exposure to a particular COG of /z/, listeners could have thus categorized /s/–/ʃ/ tokens by matching COGs: Any /s/–/ʃ/ token with a COG greater than or equal to the /z/ COG may have been categorized as /s/. Even if matching between initial consonants took place, this general auditory account mimics the predictions of spectral contrast, and also coincides with the cue covariation adaptation account: /s/ and /z/ covary in COG largely due to underlying identity in many of their spectral properties. Adaptation based on the spectral match between /s/ and /z/ is approximately equivalent to adaptation based on covariation of spectral properties between /s/ and /z/ across talkers, except that matching using representations in ASM could also allow for adaptation to nonspeech stimuli. Distinguishing between the precise predictions of spectral contrast and spectral matching in ASM remains for future research. If adaptation happened to be observed beyond the temporal window of ASM, then additional or alternative mechanisms would have to be present.

The present findings strongly implicate perceptual adaptation via a general auditory mechanism when the intervening period between auditory stimuli is quite brief. A duration of 1.3 s exceeds the limit of automatic peripheral auditory responses (Watkins, 1991); however, it is nevertheless a shorter interval than the intervening time in several medium- and long-term adaptation experiments. Adaptation to talker-specific realizations of fricatives has been observed even after several minutes or hours (e.g., Eisner & McQueen, 2005, 2006; Kraljic & Samuel, 2005). This suggests that adaptation effects exist at multiple timescales; further consideration must then be given to which mechanisms are involved at which points in time. At least with an approximately 1-s delay, general auditory influences, even if not peripheral, still dominate perception.

Nonlinguistic effects on adaptation that are originally peripheral and short-term could become centrally represented and made longer lasting by top-down influence, thus influencing speech-specific representations. Talker adaptation may then merely exist as an association between the LTAS of the talker’s speech (and any ambient noise) and the individual talker identity. A central nonlinguistic representation, such as the LTAS, could then theoretically contribute to perceptual adaptation over longer timescales (see also Alexander & Kluender, 2010). In the present study, exposure to any relevant stimuli (which could include temporally adjacent sounds) may have been integrated into the representation of the talker’s speech. Nevertheless, listeners have been shown to use top-down knowledge either about the talker or about the environment to modulate expectations about the talker’s realization of speech, indicating some amount of listener control over which auditory components can influence a speech- and talker-specific representation. Kraljic, Samuel, and Brennan (2008) have shown that if a talker has a pencil in her mouth while producing /s/, the resulting lowered spectral properties can be attributed to the pencil as opposed to being an inherent property of the talker’s speech (see also Liu, 2018; Liu & Jaeger, 2018). Moreover, listeners can adapt to multiple talkers simultaneously, further demonstrating an ability to delegate aspects of the sound stream to differing sources (e.g., Theodore & Miller, 2010; Trude & Brown-Schmidt, 2012).

### Conclusion

In the present study we investigated the mechanisms behind short-term and generalized adaptation to talker-specific spectral properties in fricative consonants. The findings revealed that auditory contrast best accounted for the observed adaptation effects when the exposure and test stimuli were adjacent to one another, with a relatively short amount of intervening time (< 2 s). Specifically, the perception of a /s/–/ʃ/ stimulus was substantially influenced by both speech and nonspeech stimuli that had a spectral manipulation in a frequency range relevant to the subsequent categorization (e.g., a /z/-initial syllable or an LTAS-matched white noise stimulus). In comparison, a cue calibration mechanism could not account for the lack of generalization from /v/ to the /s/–/ʃ/ contrast, and although cue covariation could account for generalization from /z/, it could not account for generalization from LTAS-matched white noise. Additional research will be necessary in order to address several outstanding questions, including whether auditory contrast from speech and nonspeech stimuli can persist over longer periods of time, such as several minutes or even hours, or whether linguistic mechanisms such as cue covariation must be implicated at longer timescales. Further research will also be required in order to ascertain the precise relationship between short-term and long-term adaptation mechanisms.

## Appendix

The following tables present the output from the logistic mixed-effects models fit with the brms package in R for each model (Bürkner, 2017). All effects and interactions included in the model are listed in the respective table. For each effect, the table displays the mean of the posterior distribution (Estimate), the standard deviation of the posterior distribution (Est. Error), the lower bound of the two-sided 95% credible interval (L-95% CI), the upper bound of the two-sided 95% credible interval (U-95% CI), and whether the effect or interaction is considered credible. Credibility was determined on the basis of whether the 95% CI was entirely below or above 0.

**Table 1:** Logistic mixed effects model output of /s/–/ʃ/ identification after exposure to /z/-initial syllables

Effect or Interaction	Estimate	Est. Error	L-95% CI	U-95% CI	Credible?
Intercept	0.72	0.50	– 0.30	1.70	
Condition (high vs. low)	– 1.62	0.39	– 2.41	– 0.84	✓
Vowel (/i/ vs. /u/)	2.54	0.38	1.78	3.29	✓
Speaker (“Meg” vs. “Kim”)	– 0.21	0.39	– 0.96	0.60	
Continuum step (1–9)	8.68	0.79	7.24	10.38	✓
Condition × Vowel	0.34	0.36	– 0.34	1.06	
Condition × Speaker	– 1.38	1.74	– 4.77	2.16	

**Table 2:** Logistic mixed effects model output of /s/–/ʃ/ identification after exposure to /v/-initial syllables

Effect or Interaction	Estimate	Est. Error	L-95% CI	U-95% CI	Credible?
Intercept	1.39	0.51	0.41	2.39	✓
Condition (high vs. low)	0.04	0.25	– 0.48	0.55	
Vowel (/i/ vs. /u/)	1.50	0.47	0.57	2.43	✓
Speaker (“Meg” vs. “Kim”)	– 0.02	0.24	– 0.49	0.48	
Continuum step (1–9)	6.33	0.53	5.32	7.43	✓
Condition × Vowel	– 0.56	0.40	– 1.34	0.19	
Condition × Speaker	– 0.19	1.60	– 3.35	2.97	

**Table 3:** Logistic mixed effects model output of /s/–/ʃ/ identification after exposure to white noise matched in long-term average spectrum

Effect or Interaction	Estimate	Est. Error	L-95% CI	U-95% CI	Credible?
Intercept	0.97	0.32	0.37	1.61	✓
Condition (high vs. low)	– 1.41	0.38	– 2.17	– 0.68	✓
Vowel (/i/ vs. /u/)	2.69	0.43	1.88	3.53	✓
Speaker (“Meg” vs. “Kim”)	0.72	0.36	0.03	1.41	✓
Continuum step (1–9)	9.02	0.82	7.57	10.76	✓
Condition × Vowel	0.33	0.35	– 0.40	1.02	
Condition × Speaker	0.18	1.18	– 2.13	2.59	

**Table 4:** Logistic mixed effects model output of /s/–/ʃ/ identification from all three experiments: Exposure to /z/, exposure to /v/, and exposure to white noise

Effect or Interaction	Estimate	Est. Error	L-95% CI	U-95% CI	Credible?
Intercept	0.29	0.44	– 0.59	1.16	
Condition (high vs. low)	– 0.48	0.31	– 1.10	0.13	
Vowel (/i/ vs. /u/)	2.11	0.33	1.50	2.79	✓
Speaker (“Meg” vs. “Kim”)	0.18	0.26	– 0.34	0.68	
Continuum step (1–9)	7.09	0.67	5.86	8.48	✓
Condition × Vowel	– 0.06	0.45	– 0.92	0.85	
Condition × Speaker	– 0.76	1.60	– 3.97	2.45	

**Table 5:** Logistic mixed effects model output of /s/–/ʃ/ identification after exposure to alternating /z/-initial syllables and white noise that differed in center of gravity (COG)

Effect or Interaction	Estimate	Est. Error	L-95% CI	U-95% CI	Credible?
Intercept	0.68	0.41	– 0.10	1.49	
Condition (high vs. low)	– 1.54	0.31	– 2.15	– 0.94	✓
Vowel (/i/ vs. /u/)	2.40	0.39	1.65	3.17	✓
Speaker (“Meg” vs. “Kim”)	0.18	0.18	– 0.17	0.54	
Continuum step (1–9)	7.93	0.37	7.22	8.67	✓
Experiment 1 (/v/)	1.52	1.18	– 0.88	3.78	
Experiment 2 (noise)	0.57	1.08	– 1.60	2.61	
Condition × Vowel	0.32	0.36	– 0.40	1.01	
Condition × Experiment 1	2.99	0.90	1.26	4.79	✓
Condition × Experiment 2	0.52	0.88	– 1.19	2.29	
Vowel × Experiment 1	– 1.37	1.10	– 3.53	0.84	
Vowel × Experiment 2	0.24	1.08	– 1.94	2.37	
Condition × Vowel × Experiment 1	– 2.05	1.07	– 4.17	0.06	
Condition × Vowel × Experiment 2	0.08	1.08	– 2.07	2.19	

Note that the effect of condition refers to the COG of the speech stimulus.

## Footnotes

1 These data are available as supplemental material to McMurray and Jongman (2011): doi:10.1037/a0022325.supp.

2 Differences in the mid to low frequencies of the /s/ and /ʃ/ spectra likely also contribute to identification. Our focus on the upper region of the spectrum is based on the fact that the manipulated high- and low-COG /z/-initial stimuli primarily differed above ~6 kHz and otherwise were similar in their spectral makeup (see Fig. 3). Any observed differences in categorization after exposure to the /z/-initial stimuli or LTAS-matched white noise were thus attributed to differences in the distribution of energy in this upper frequency range.

3 The fricative onset and offset were manually segmented and corresponded to the frication period, as indicated in the waveform and spectrogram. Prevoicing was not included in this interval, and the fricative offset was marked at the F2 onset in the following vowel. COG was measured from a multitaper spectrum (Blacklock, 2004) applied to the middle 50% of the sibilant fricative after band-pass filtering between 550 and 10000 Hz. The high-pass filter minimized strong influences of voicing in the measurement, whereas the low-pass filter approximated the relevant upper limit utilized in the perception of sibilant fricatives (based on Stelmachowicz, Pittman, Hoover, & Lewis, 2001).

4 The COG of the final burst could potentially affect the perception of /s/ and /ʃ/ under any of the accounts considered. Between participants, the bursts were identical for the high- and low-COG /z/ conditions for a given speaker (Meg or Kim). Each participant, however, did hear two different sets of bursts: one from Meg and one from Kim. If a speaker’s realization of /t/ did contribute substantially to /s/–/ʃ/ perception, we would expect to find a main effect of speaker (e.g., Kim’s /t/ would lead to more /s/ responses), which we did not find (see the Exp. 1 Results section).

5 To ensure that the heterogeneity of language backgrounds did not disproportionately influence the response pattern, we ran an additional model with a structure identical to the primary model but using data from the monolingual speakers alone (*N* = 23 participants; five participants omitted). The overall pattern of results was the same, and importantly, the effect of condition remained credible (β = – 1.69, 95% CI: [– 2.65, – 0.78]).

6 An additional model was run on data from the monolingual speakers alone (*N* = 25 participants; three participants omitted). The pattern of results remained the same, and the COG condition did not compellingly influence /s/–/ʃ/ categorization among these speakers (β = 0.08, 95% CI: [– 0.47, 0.64]).

7 If the final burst contributed substantially to perception of the /s/–/ʃ/ stimuli, then we would expect to find a difference in the response patterns between the /z/ and noise exposure experiments. As we discussed in the Experiment 2 Procedure section, no remarkable difference was found between these two experiments.

8 An additional model that excluded the two multilingual participants yielded the same pattern of results. The effect of condition remained credible among the monolingual participants (β = – 1.15, 95% CI: [– 1.81, – 0.55]).

9 The locations of the response curves were comparable for both for the /z/-initial and LTAS-matched noise stimuli, but we cannot conclusively state that the direction of the shift was the same.

10 The same pattern of results was found with a model using data from just the monolingual participants (*N* = 21 participants; seven participants omitted). Critically, the effect of the speech COG was not reliably observed (β = – 0.06, 95% CI: [– 0.63, 0.52]).
